# Study of Histopathological and Molecular Changes of Rat Kidney under Simulated Weightlessness and Resistance Training Protective Effect

**DOI:** 10.1371/journal.pone.0020008

**Published:** 2011-05-23

**Authors:** Ye Ding, Jun Zou, Zhili Li, Jijing Tian, Saed Abdelalim, Fang Du, Ruiping She, Desheng Wang, Cheng Tan, Huijuan Wang, Wenjuan Chen, Dongqiang Lv, Lingling Chang

**Affiliations:** 1 Department of Veterinary Pathology, Key Laboratory of Zoonosis of Ministry of Agriculture, College of Veterinary Medicine, China Agricultural University, Beijing, China; 2 National Animal Protozoa Laboratory, College of Veterinary Medicine, China Agricultural University, Beijing, China; 3 State Key Laboratory of Space Medicine Fundamentals and Application, China Astronaut Research and Training Centre, Beijing, China; Universidade de Sao Paulo, Brazil

## Abstract

To explore the effects of long-term weightlessness on the renal tissue, we used the two months tail suspension model to simulate microgravity and investigated the simulated microgravity on the renal morphological damages and related molecular mechanisms. The microscopic examination of tissue structure and ultrastructure was carried out for histopathological changes of renal tissue morphology. The immunohistochemistry, real-time PCR and Western blot were performed to explore the molecular mechanisms associated the observations. Hematoxylin and eosin (HE) staining showed severe pathological kidney lesions including glomerular atrophy, degeneration and necrosis of renal tubular epithelial cells in two months tail-suspended rats. Ultrastructural studies of the renal tubular epithelial cells demonstrated that basal laminas of renal tubules were rough and incrassate with mitochondria swelling and vacuolation. Cell apoptosis in kidney monitored by the expression of Bax/Bcl-2 and caspase-3 accompanied these pathological damages caused by long-term microgravity. Analysis of the HSP70 protein expression illustrated that overexpression of HSP70 might play a crucial role in inducing those pathological damages. Glucose regulated protein 78 (GRP78), one of the endoplasmic reticulum (ER) chaperones, was up-regulated significantly in the kidney of tail suspension rat, which implied that ER-stress was associated with apoptosis. Furthermore, CHOP and caspase-12 pathways were activated in ER-stress induced apoptosis. Resistance training not only reduced kidney cell apoptosis and expression of HSP70 protein, it also can attenuate the kidney impairment imposed by weightlessness. The appropriate optimization might be needed for the long term application for space exploration.

## Introduction

The necessity and advantages of human in the exploration of space have been highlighted by many successful space missions over the past 50 years [1]. But as human space travel is more feasible in the twenty-first century, the health and safety of space explorers become the most concerned question. Because spatial experiments are demanding and expensive, there are several experimental models on the Earth to simulate weightlessness. Rat-tail suspension model was used by National Aeronautics and Space Administration (NASA) to simulate weightlessness on the Earth under laboratory conditions. It was firstly introduced and used by Morey-Holton [2] and later improved by Morey-Holton and Globus [3]. The tail suspension model has been used in studies of muscle atrophy and osteoporosis in microgravity states [4], [5], [6], [7]. Also the tail suspension model is considered to be a model to study the effect of body fluid shift which occurred in weightlessness condition [8].

Under microgravity condition, there was a cephalic shift of fluids in human [9]. Kidney, the main organ that participates in maintaining body fluid and acid-base balance, significantly contributes to the control of vascular volume and excretes metabolites. A number of studies have demonstrated that renal function was influenced during and immediately following spaceflight. Natochin et al found that postflight urine osmolality of the astronaut was always lower than preflight levels for any given urine flow rate [10]. In addition, Gazenko and Natochin et al demonstrated that the ability to excrete a fluid load appeared to be impaired following spaceflight [10], [11]. Zorbas et al studied the effect of weightlessness on rat kidney and they observed an increased weight of kidneys and marked morphological changes in the structure of nephrons, particularly in collecting tubules [12].

Although there are a few studies that plainly show morphological changes and cell apoptosis of kidney under simulated microgravity, the in-depth histopathological damages and the apoptotic mechanisms are not fully investigated. Furthermore, a suggestive approach to minimize the kidney damage under simulated microgravity has been an emerging arena.

## Results

### Body weight and renal index

**Figure 1:**
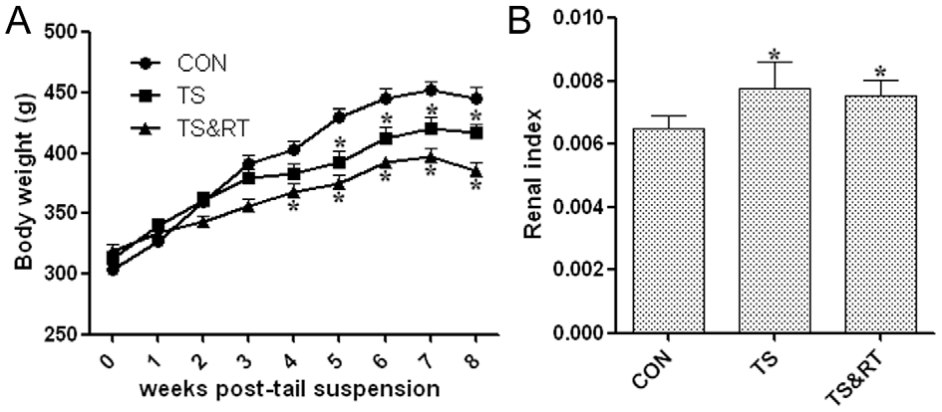
Body weight and renal index under microgravity condition. A: Dynamic changes of rats body weight in control group (CON), tail-suspended group (TS) and, tail-suspended and resistance training group (TS&RT). B: Renal index was obtained by dividing total left and right kidney weight to the body weight of euthanized rats. Data are shown as means±SD. * Significant *P* values<0.05.

Several parameters such as body weight and renal index were compared between different groups under the influence of weightlessness. The initial body weight of control group, TS group and TS&RT group was 304.0±8.7 g, 312.6±5.7 g and 318.8±5.2 g, respectively. No significant differences of initial body weight were recorded (Fig. 1 A). The rats of TS group were significantly lighter than those of the control group during the tail suspension experiment and the weight difference between the two groups became more significantly apparent after 8 weeks in tail-suspended rats (Fig. 1A). 3 weeks after the tail suspension,the weight of rats in the TS&RT group was lighter compared with that in the control group or the TS group, and they maintained this reduced body weight for the rest of the experiment (Fig. 1A).

Renal index was obtained by dividing total left and right kidney weight to the body weight of euthanized rats. The renal index of rat in TS group was significantly greater than that in control group possibly due to rat kidney swelling in simulated weightlessness (Fig. 1B). However, no significant difference of the renal index was detected between TS group and TS&RT group.

### The histopathological observation of the kidney with Light microscopy

**Figure 2:**
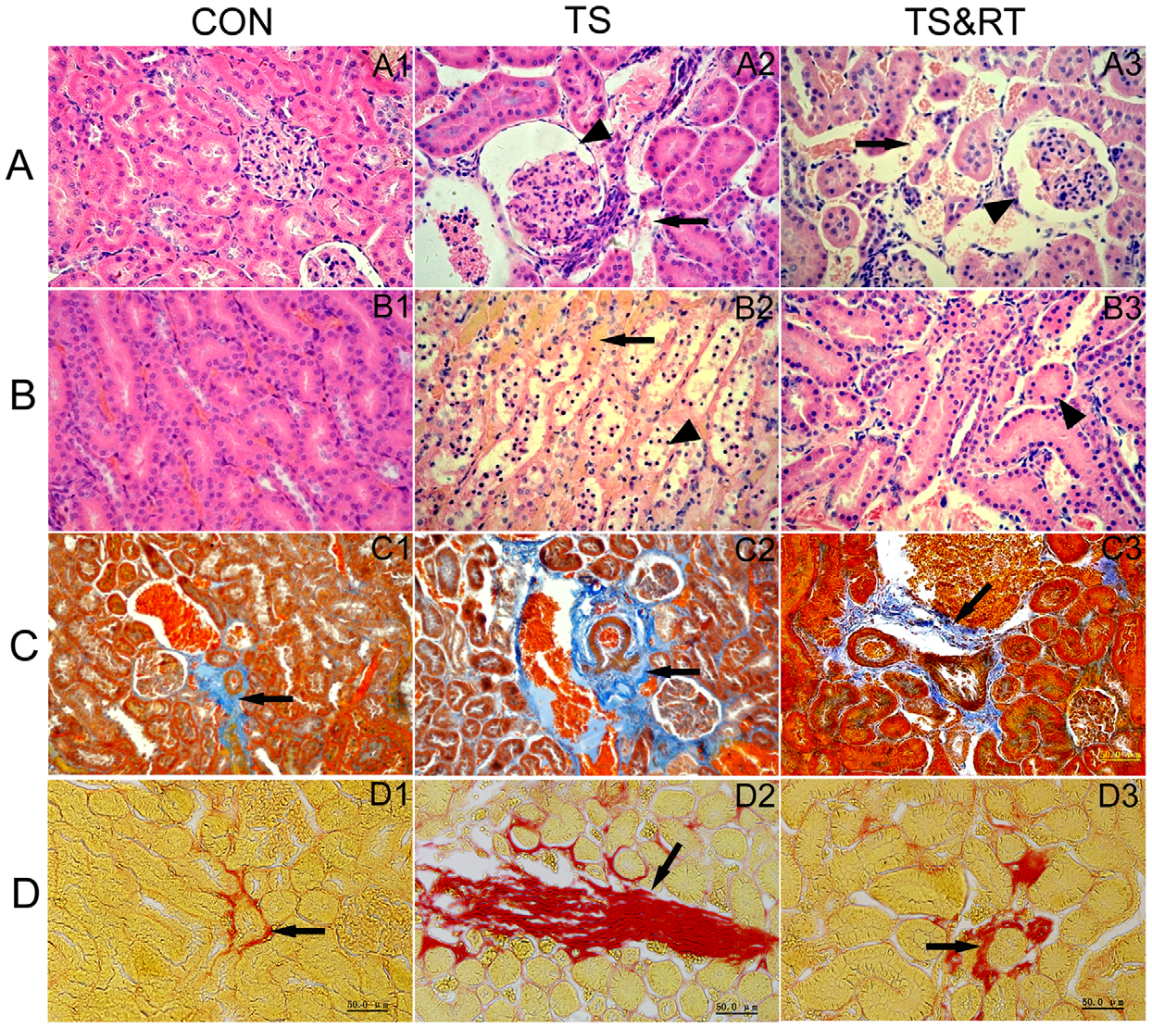
Histopathological analysis of rats' kidneys paraffin sections stained with hematoxylin and eosin (HE), mallory's trichrome staining and sirius red staining. A: Morphological changes of renal cortex: The normal structure of rats' kidney in the control group (A1). In TS group, rat's renal cortex showed glomerular atrophy, distension of the renal glomerulus capsular space (arrowhead) and interstitial edema (arrow, A2). In TS&RT group, rat's renal cortex displayed moderately glomerular atrophy (arrowhead) and interstitial congestion (arrow, A3). B: Morphological changes of renal medulla: The normal structure of rats' kidney in the control group (B1). In TS group, rat's renal medulla abundantly illustrated degeneration and necrosis of renal tubular epithelial cells (arrowhead), interstitial congestion (arrow), and protein and cellular cast in nephric tubules (B2). In TS&RT group, rat's renal medulla showed scattered degeneration and necrosis of renal tubular epithelial cells (arrowhead, B3). C&D: Mallory's trichrome (C) and Sirius Red (D) stained paraffin sections displayed different levels of proliferation of kidney fibrous tissue in CON(C1,D1), TS(C2,D2) and TS&RT groups (C3,D3)respectively. Collagen fibers were stained blue with Mallory's trichrome and red with Sirius Red respectively (arrow).

The results obtained by HE staining showed that in the rat kidney of TS group there were obvious pathological lesions characterized by glomerular atrophy, extension of the renal glomerulus capsular space, serious renal tubular epithelial cells degeneration and necrosis, abundant protein exudation in the renal tubular lumen, renal interstitial edema and haemorrhage (Fig. 2A2&B2). In the rats' kidney of TS&RT group, these pathological damages were lighter than those of TS group, although glomerular atrophy, renal interstitial edema and congestion, renal tubular epithelial cells degeneration and necrosis were also sporadically observed. The severity of pathological damage was reduced (Fig. 2A3&B3). No pathological changes were found in the kidney of control group (Fig. 2A1&B1).

Mallory's trichrome staining was widely used to detect the fibroplasia, although it could stain some matrix components [13]. In the present study, abundant fibroplasia was observed in the kidney of TS group by Mallory's trichrome staining, as compared to control group that few fibrous tissues around the blood vessel was presented (Fig. 2C1&C2). In the TS&RT group where the rats received resistance training, fibroplasia in the kidneys was observed to be reduced (Fig. 2C3) compared to TS group. The relative degree of fibroplasia in these groups was further confirmed by Sirius Red staining which has been used to detect collagen (Fig. 2D). Semi-quantitative analysis of staining intensity showed that it nearly increased to 4-fold in TS group compared with the control group, and it was observed that fibroplasia in TS&RT group decreased to 0.6-fold of that in TS group (Table 1).

**Table 1:** Semi-quantitative analysis of fibrosis with Mallory's trichrome and Sirius Red staining.

	Mallory's trichrome staining (%)	Sirius Red staining (%)
CON	3.25±0.965	2.38±0.332
TS	13.3±3.05**	9.49±1.67**
TS&RT	8.94±2.82*	5.47±1.01*

Each value represents the percentage of mean area density of collagen in each group. n = 150 fields (10 rats) for each value.

*P<0.05,

**P<0.01.

### Transmission electron microscopic observation of the kidney under simulated microgravity environment

**Figure 3:**
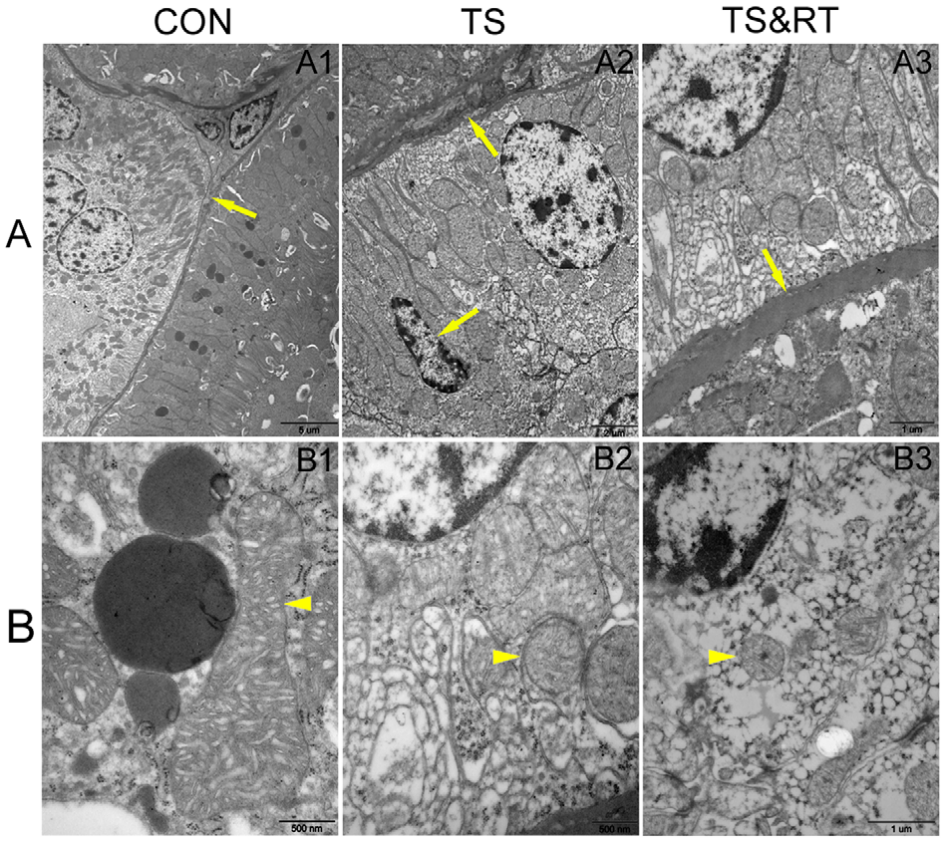
The transmission electron micrographs of kidney tissue in CON, TS and TS&RT groups. A1 and B1: The basal lamina of renal tubules was intact (arrow) and there were abundance of mitochondria of spherical or elongated shape with mitochondrial cristae in the CON group (arrowhead) A2 and B2: The basal laminas of renal tubules were rough and incrassate (arrow), the proximal tubules and distal tubules had numerous dead cells (arrow). Mitochondria in electron-dense cytoplasm of the cell were swollen with thin cristae (arrowhead) in the TS group. A3 and B3: The incrassate of renal tubules basal lamina (arrow) with mitochondria swelling (arrowhead) in renal tubule epithelia of TS&RT group.

To further investigate renal injury in weightlessness condition, the ultrastructural changes of rat kidney in the three groups were comparatively analyzed by transmission electron microscopy. In control group, there were no apparent damages as judged by intactness of the basal lamina of renal tubules of rats' kidney tissue. The spherical and elongated mitochondria were abundant with mitochondria cristae and the cisternae of endoplasmic reticulum were smooth. High, thin and parallel microvilli were observed on the apical surface of renal tubular epithelial cells (Fig. 3A1&B1).

In TS group, the basal laminas of renal tubules were rough and incrassate, and dead cells were found in proximal tubules and distal tubules. Mitochondria in electron-dense cytoplasm of the cell were swollen with thin cristae. The endoplasmic reticulum was expansive and the nucleus was shriveled (Fig. 3A2&B2). In TS&RT group, the degree of mitochondria swelling and vacuolation was relieved, although some dark cells with dark shrivelled nuclei were observed in the renal tubular epithelium (Fig. 3A3&B3). The results demonstrated that tail-suspended rats produced severe pathological damage in kidney and pathological lesions induced by the weightlessness could be reduced through resistance training.

### The expression of PCNA and HSP70 was associated with pathological changes of the kidney under simulated weightlessness environment

**Figure 4:**
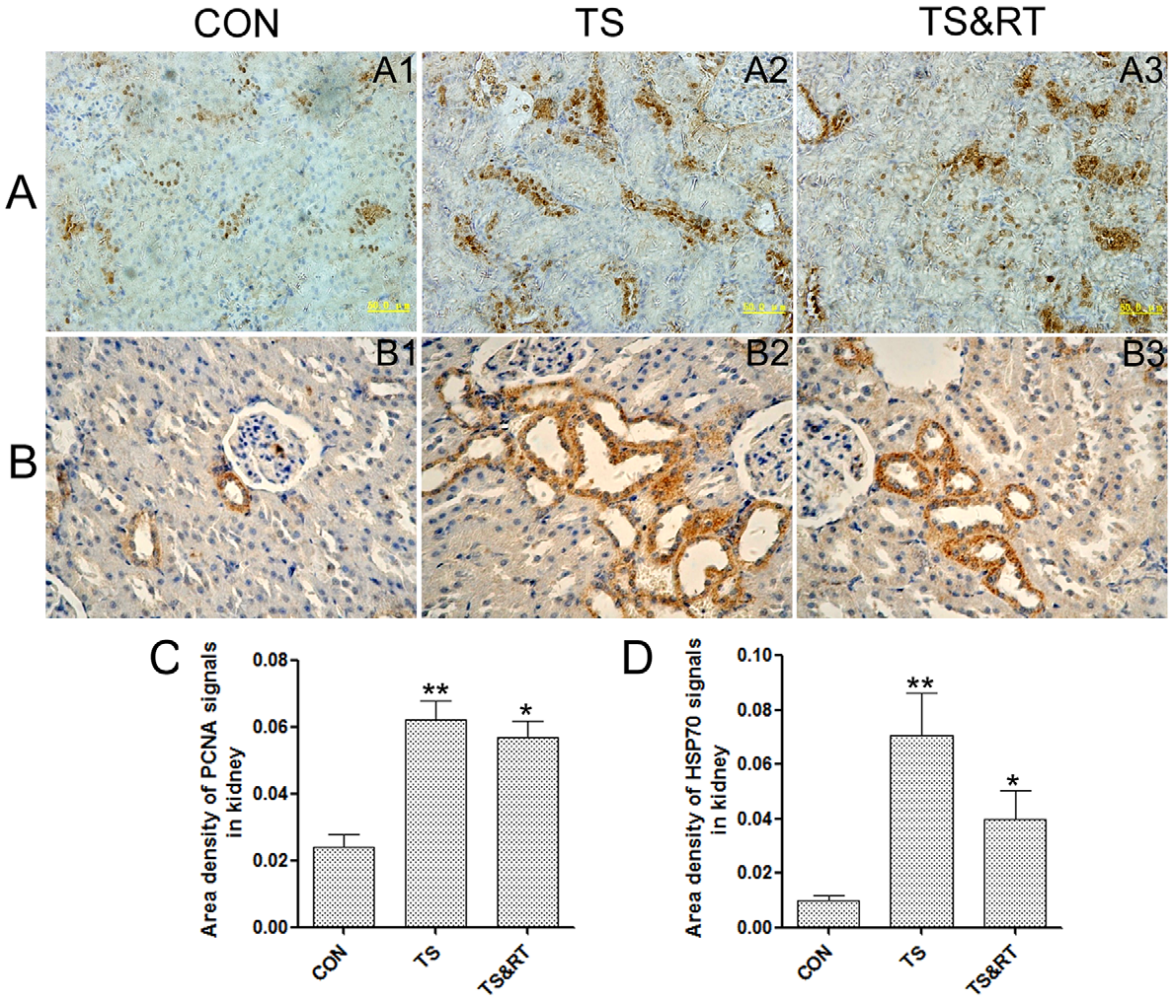
Immunohistochemical staining of PCNA and HSP70 proteins in rats' kidneys. A: Histological sections of kidneys in CON, TS and TS&RT groups (A1–A3) respectively were stained with anti PCNA serum. B: Histological sections of kidneys in CON, TS and TS&RT groups (B1–B3) respectively were stained with anti HSP70 protein serum. C and D: Semi-quantitative analysis of PCNA and HSP70 proteins expression in kidneys with immunohistochemical staining. Data are shown as means±SD. *P significant *p* values<0.05 and **P significant *p* values<0.01.

Proliferating cell nuclear antigen (PCNA), the component of the replication and repair machinery, plays an essential role in nucleic acid metabolism [14]. The increased expression of PCNA implied that cells were under damaged condition and required compensatory proliferation response [15]. The immunohistochemical results showed that the PCNA was distributed diffusely throughout the tubules of the kidney in TS group (Fig. 4A2), while few PCNA-positive cells were found in the renal tubular epithelium in the control rats (Fig. 4A1). In TS&RT group, fewer PCNA-positive cells were found in renal tubular epithelium compared with those of TS group (Fig. 4A3). We could not find PCNA-positive cells in the glomeruli. The semi-quantitative analysis showed that the PCNA protein expression in the kidney of TS group was nearly a 2.5-fold higher compared to control group (Fig. 4C). The moderate decrease of PCNA protein expression in TS&RT group compared with TS group was observed (Fig. 4C). The change of PCNA protein expression was consistent with the pathological damage in rats' kidney.

Moderate expression of HSP70 had a protective role under stress condition, but when the HSP70 was overexpressed or released from the cell, it was a danger signal that might trigger cell necrosis [16], [17], [18], [19]. Immunohistochemical study showed that the TS group HSP70 protein was richly expressed in the renal tissue of rats (Fig. 4B2) with increase for about 7-fold (Fig. 4D) compared with that in the control group which showed least or modest HSP70 protein expression in rats' kidney (Fig. 4B1). Some HSP70 proteins released from the kidney cells and distributed in kidney tubules and renal interstitium (Fig. 4B2). This implies that HSP70 protein might be one of the necrosis-inducing agents in renal tissue under weightlessness. The HSP70 protein expression in the kidney of TS&RT group was remarkably lower than that of TS group following resistance training, although it was still significantly higher than that of control group (Fig. 4B3). Semi-quantitative analysis of HSP70 expression in TS&RT group showed that a 4-fold increase and a 0.5-fold decrease was found as compared with that in the control and TS groups respectively (Fig. 4D).

### Up-regulation of apoptosis-regulating proteins in TS rat kidneys

**Figure 5:**
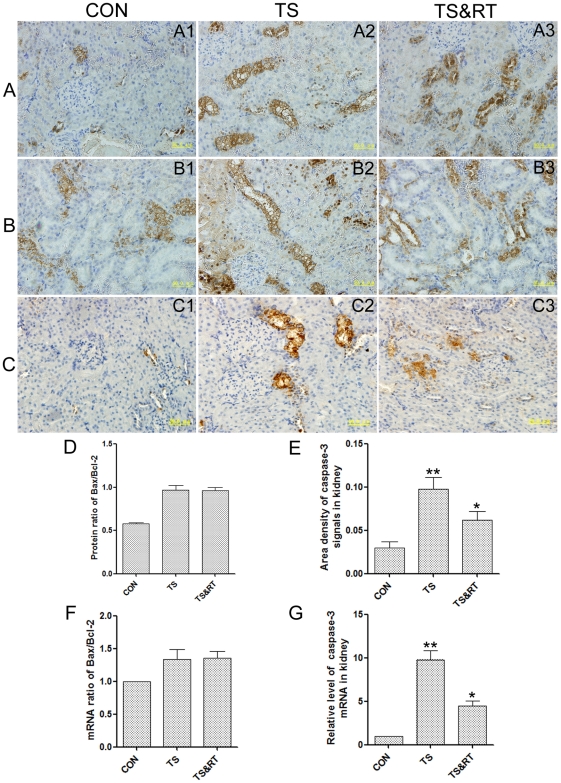
Immunohistochemical staining and real-time PCR analysis of apoptosis. A: Histological sections of kidneys in CON, TS and TS&RT groups (A1–A3) respectively were stained with anti Bax protein serum. B: Histological sections of kidneys in CON, TS and TS&RT groups (B1–B3) respectively were stained with anti Bcl-2 protein serum. C: Histological sections of kidneys in CON, TS and TS&RT groups (C1–C3) respectively were stained with anti caspase-3 protein serum. D: The protein ratio of Bax/Bcl-2 is obtained by dividing signal intensity of Bax positive substance and Bcl-2 positive substance. E: Semi-quantitative analysis of caspase-3 protein with immunohistochemical staining. F and G: mRNA ratio of Bax/Bcl2 and relative mRNA levels of caspase-3 in CON, TS and TS&RT rats' kidney A total of 10 samples for each group were used, and each sample was run in triplicate for real-time PCR. Data are shown as means±SD. *P significant *p* values<0.05 and **P significant *p* values<0.01.

The Bax and Bcl-2 are pro-apoptotic and anti-apoptotic proteins respectively and the Bax/Bcl-2 ratio (the apoptotic index) reflected a cell's vulnerability to apoptosis [20]. In the present study, the Bax and Bcl-2 proteins were measured as an area density of Bax or Bcl-2 positive substance by immunohistochemical staining. The ratio of Bax/Bcl-2 was obtained by dividing area density of Bax positive substance over Bcl-2 positive substance. The upregulation of both Bax and Bcl-2 protein expression in 8 weeks tail-suspended rats was detected compared with that of the control group (Fig. 5A&B). The apoptotic index was higher compared with those in the control group by semi-quantitative analysis (Fig. 5D). No significant difference in the Bax and Bcl-2 expression was detected between the TS and TS&RT group (Fig. 5D). These results were further confirmed by the analysis of Bax and Bcl-2 gene expression using real-time PCR (Fig. 5F).

The partial or total responsibility of caspase-3 for the proteolytic cleavage of several cellular proteins in various systems as a key executioner was reported by Cohen [21] and Nunez et al [22]. The immunohistochemical study showed that, in TS group the caspase-3 protein was profusely expressed in the rats' renal tissue (Fig. 5C2), with a 3.2-fold increase (Fig. 5E) compared to the control group with modest or low caspase-3 expression (Fig. 5C1). In the TS&RT group, the expression of caspase-3 reduced to 0.33-fold compared with TS group by semi-quantitative immunohistochemical analysis (Fig. 5E). The results were consistent with mRNA expression analyzed by real-time PCR (Fig. 5G), suggesting that apoptosis might be involved in the impairment of kidney under simulated weightlessness.

### Up-regulation of ER chaperone GRP78 in kidneys of tail suspended rats

**Figure 6:**
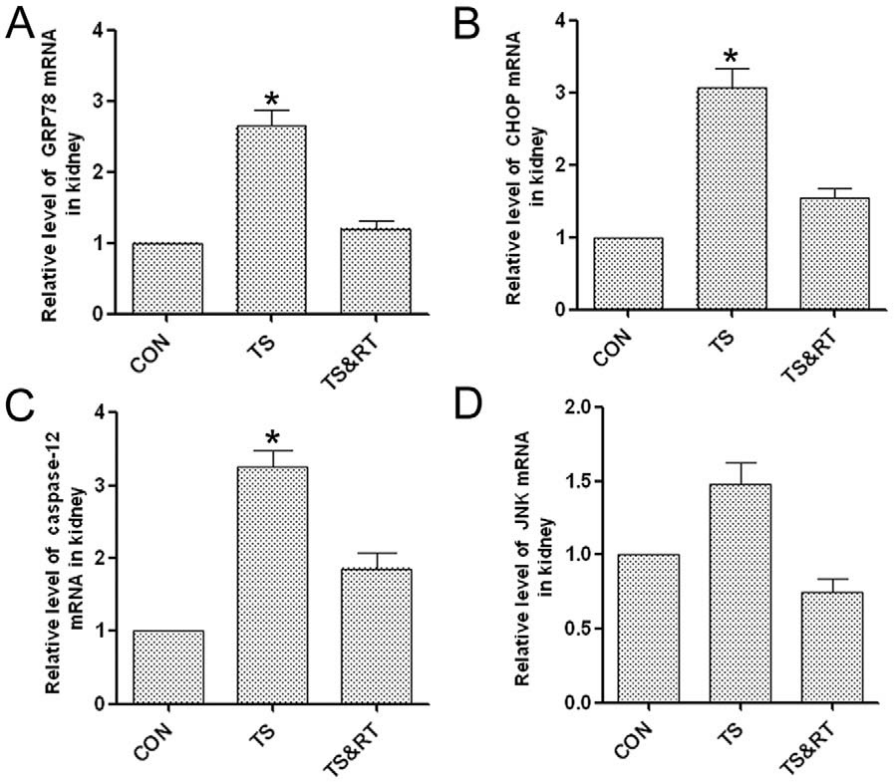
Relative mRNA levels of GRP78(A) and CHOP(B), caspase-12(C), and JNK(D) in CON, TS and TS&RT rats' kidney. A total of 10 samples for each group were used, and each sample was run in triplicate for real-time PCR. Data are shown as means±SD. *P significant *p* values <0.05 and **P significant *p* values <0.01.

Many factors, such as hypoxia, oxidative injury, viral infection, etc., can induce ER stress. GRP78, one important molecular chaperone localized in ER, has been used extensively as an indicator for the induction of ER stress [23], [24]. As shown in Fig. 6A, real-time PCR study showed that GRP78 mRNA in the renal tissue of TS rats was approximately 2.6-fold higher compared to the control group. By Comparing the GRP78 gene expression, we found that GRP78 mRNA level in the TS&RT group was significantly lower than that in the TS group (Fig. 6A).

### Involvement of the ER-associated apoptosis pathways

**Figure 7:**
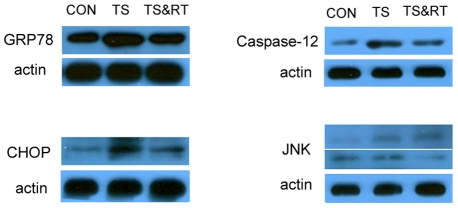
Western blot analysis of the expression of GRP78, caspase-12, CHOP, and JNK in the kidneys of CON, TS and TS&RT groups.

ER stress is a double-edged weapon for cell survival. Severe and prolonged ER stress activates three unique pathways that lead to cell death through apoptosis. CHOP, JNK, and caspase-12 were the key molecules in the three branches of ER-associated apoptosis pathways [25], [26], [27], [28], [29], [30]. Our studies showed that under simulated weightlessness, the renal cells undergo apoptosis and ER stress, therefore our focus will be directed to investigate whether the apoptosis is associated with ER stress. The gene expressions of CHOP and caspase-12 were significantly increased in the kidney of TS group (Fig. 6B&C). However, the expression of JNK did not show any remarkable modifications between the TS and control group (Fig. 6D). In the TS&RT group, the gene expression of CHOP and caspase-12 was remarkably reduced compared with TS rats (Fig. 6B&C). Furthermore, western blot analysis showed the same trend of GRP78, CHOP, Caspase-12 and JNK in protein level (Fig. 7). These finding clearly demonstrated that CHOP and caspase-12 pathways, which happened in prolonged ER stress-induced apoptosis, were activated in TS group and RT could reduce ER stress-induced apoptosis.

## Discussion

A number of studies have demonstrated that renal function is influenced by the environment of weightlessness [31], [32], [33], [34]. Using short-term space flight experiment, some reporters suggested that space environment might affect in kidney development [35]. With the development of technology, people can stay in the space for a longer period. It is necessary to investigate the detailed effect of long-term microgravity on renal morphological changes. In the present investigation, 8 weeks tail suspension model was designed to simulate weightlessness condition and demonstrated that the pathological changes such as glomerular atrophy, degeneration and necrosis of renal tubular epithelial cells that were associated with long-term weightlessness were severe compared to the short-term weightlessness condition. Some of those pathological changes were not reversible, and could seriously affect the kidney function. Therefore, precautions must be raised to protect kidney damages of astronauts, which might be occurred as a result of weightlessness condition during long-duration missions in space.

Accumulating evidences demonstrated that Endoplasmic reticulum (ER) could directly initiate pathways to caspase activation and apoptosis [36]. The involvement of ER stress in a wide range of pathological circumstances such as diabetes mellitus, ischemic injury, cancers, inflammation, infection, toxicity of chemicals and metals, and psychotic diseases was studied by Kim et al [37]. Furthermore, more evidences showed that albumin could induce ER stress and apoptosis in renal proximal tubular cells in vitro [38], [39] and ER stress was also involved in podocyte injury induced by excessive protein accumulation [40], [41]. In the present study, enriched protein exudation in the renal tubular lumen and swollen endoplasmic reticulum was observed in kidney of TS rats, which was a positive indication for the involvement of ER stress in the apoptosis induced by tail suspension. Furthermore, the expression of ER molecular chaperone and ER-associated apoptotic proteins was analyzed using real time PCR and western blotting. The results illustrated that the considerable higher level of gene expression in TS rats was detected as compared to the control group, indicating that kidney ER stress was associated with apoptosis in renal proximal tubular cells induced by simulated weightlessness. However, the clear mechanism remains to be further studied.

Countermeasures were developed to prevent microgravity-induced musculoskeletal loss and provide successful post-flight rehabilitation [42]. There were many forms of resistance exercise [43], [44], [45]; resistance training (RT) using a weight lifting apparatus, as one countermeasure, appeared to be promised in ameliorating muscle and bone loss in microgravity. Here we used one weight lifting apparatus to guide rats performing the resistance training. However, this device requires the rats to maintain standing position, which could induce the rat body fluid shift and change microgravity state of the rat. Therefore further studies need to improve this device to satisfy the requirement of the rats to perform RT under tail suspension state, and the appropriate optimization of the characteristics of RT such as the maximum weight, the number of repetitions for each set, etc. should also be considered.

Many studies have demonstrated that RT could increase nitrogen retention and protein synthesis, ameliorates losses of muscle mass and its function, and effectively alleviate kidney disease [46], [47], [48]. The RT that was accessible to astronauts attracted our attention for addressing its protective effects on kidney under simulated weightlessness. Our results showed that the kidney impairment imposed by weightlessness condition in TS&RT group is less severe than that in TS group. To determine whether the less severity of impairment in TS&RT group is due to the RT or body fluid shift induced by standing position in this weight lifting apparatus, addition group in which the rats were kept in standing position but did not perform RT was included in this experiment. The results obtained from HE staining showed that pathological lesions in the rat kidney of this group were comparable to that in the TS group (Data not shown), demonstrating that resistance training was responsible for the attenuation of kidney impairment caused by weightlessness condition.

In summary, our study comprehensively investigated the pathological changes of rat kidney imposed by simulated weightlessness with in-depth insights to the correlation of ER stress-induced apoptosis, and overexpression of HSP70 protein with the pathological damages. The development of effective measures using RT with appropriate optimization should be considered to protect crew member of a spacecraft from the pathological damages.

## Materials and Methods

### Tail suspension (TS) and Resistance training (RT)

Male Wistar rats weighting 290±20 g(8-week-old) were obtained from Experimental Animal Center, Academy of Military Medical (certificate number: 038695). These rats were separately caged in an air-conditioned room maintained at 23°C with a cycle of 12 h of light and 12 h dark. These rats were randomly assigned to three groups of 10 rats each; control group (without tail suspension for 8 weeks), tail suspension (TS) group, and tail suspension and resistance training (TS&RT) group. The rats of control group were housed in standard cages in the same room as tail-suspended groups. TS group was achieved by using of a tail harness to suspend the hindlimbs above the floor of the cage according to the method of Wronski and Morey-Holton [49]. TS&RT group was achieved according to Klitgaard et al with some modifications [50]. Briefly, the RT device contained an electrical stimulus apparatus, an indicator lamp and a special orthostatic tube. Rats were strapped into the orthostatic tube to let the rat remain standing and the rat's tail was attached to an electrode which could deliver electrical stimulus to initiate squats by a manual switch. The indicator lamp was used to guide the rat to lift weights. The rat lifted the weights by conditioned reflex with 2 seconds interval according to light-on and light-off signals. If the rat did not follow the instruction of indicator lamp, the device would send electrical pulse with voltage of 10 V and pulse width of 0.3 s to stimulate the rat to exercise. The rats took four sets (12 repetitions for each set) at 65% to 75% of 1RM (the maximum weight lifted by rat with the squat-training apparatus), and between each set the rats were allowed to rest with a standing position in the apparatus for 90 s. The resistant training performed five times per week for 8 weeks. Prior to the application of tail suspension, the animals from the TS&RT group were accommodated to RT by using the above device to lift 50 g plus their own body weight. This RT was conducted for 30 min per day for 3 days to allow the animals accommodating to luminous stimulus and performing the squat movement. All experimental procedures were approved by the China Astronaut Research and Training Center Laboratory Animal Care Committee rules and the Institutional Animal Care and Committee of China Agricultural University (The certificate of Beijing Laboratory Animal employee, ID: 15883).

### Sampling

Two months post-tail suspension, rats' body weight were measured, kidneys were removed quickly and washed thoroughly with normal saline to remove the residual blood after euthanasia and then weighed and processed for biochemical and morphological studies.

### Histopathological examinations

#### 1. Light microscopy (LM)

Kidneys were collected and fixed in 2.5% (v/v) glutaraldehyde-polyoxymethylene solution immediately after rats' euthanasia. The tissue samples were dehydrated and embedded in paraffin wax. Serial paraffin sections (4 um) were obtained and kept at 37°C for more than 12 h. The sections were immersed in three consecutive washings in xylol for 5 min to remove paraffin, and then hydrated with five consecutive washings with alcohol in descending order 100, 95, 80, 70, 50% and deionized water respectively. The histological paraffin sections were then treated with HE staining. Changes in organizational structure were visualized using a light microscope. Other histological paraffin sections were stained with Mallory's trichrome or Sirius Red to study the kidney fibrosis. Collagen fibers were stained blue with Mallory's trichrome and red with Sirius Red respectively. Three fields per section and five sections per rat were analyzed. Each analyzed field was chosen randomly and the collagen in kidneys was measured using Motic Med 6.0 CMIAS Image Analysis System (MOTIC CHINA GROUP CO., LTD). The area density which represented the collagen staining intensity was calculated as the ratio between the stained area and the total analyzed field.

#### 2. Transmission electron microscopy (TEM)

For transmission electron microscopy (TEM) the kidney samples were cut into pieces (2×2 mm) and fixed in 2.5% (v/v) glutaraldehyde-polyoxymethylene solution for 6–8 h at 4°C. They were washed and post fixed in 2% OsO4 for 1 h at 4°C. The tissue was dehydrated through ascending grades of ethanol and embedded in araldite CY212. Ultra thin sections (60–70 nm) were cut and stained with uranyl acetate and alkaline lead citrate. Sections were visualized under a JEM 100CX transmission electron microscope.

### Immunological assays

The kidney tissue samples collected for histology were subjected to immunohistochemical analysis for examination of heat shock protein 70 (HSP70), Proliferating Cell Nuclear Antigen (PCNA), Bax/Bcl-2 and caspase-3 protein. The histological sections (4 µm) were immersed in citrate buffer solution (0.01 M, pH6.0) and heated at 120°C in autoclaved sterilizer for 10 min, naturally cooled for 30 min, and then immersed in 3% aqueous hydrogen peroxide (H_2_O_2_) for endogenous peroxidase ablation at room temperature for 30 min. The following steps were executed in a moist chamber and this procedure has been exclusively described by Liu et al [51]. Briefly, the histological sections were washed in PBS, quenched with blocking buffer (Zymed Laboratories Inc., San Diego, USA). Sections were sequentially treated with primary antibody, biotinylated secondary antibody and horseradish peroxidase-labeled avidin chain working fluid (Beijing Zhong Shan Golden Bridge Biotechnology Co., Ltd., Beijing, China). Finally, the tissue sections were counterstained with hematoxylin, dehydrated, cleared and mounted with neutral gums. In parallel, tissue specimens in which the primary antibody was replaced by PBS served as negative control. Specificity was established by demonstrating the loss of immunoreactivity in matched tissue sections.

The positive signal of HSP70, PCNA, Bax/Bcl-2 and caspase-3 proteins was brown or yellow granular mass that could be used to trace and measure the aforementioned proteins. The positive signals of these proteins were measured using Motic Med 6.0 CMIAS Image Analysis System (MOTIC CHINA GROUP CO., LTD). A total of 150 fields per rat (three fields per section and five sections per rat) were chosen randomly and analyzed. The area density which represented the positive staining intensity was calculated as the ratio between the stained area and the total analyzed field.

### Real-time quantitative RT-PCR

Total RNA was extracted from rat kidney tissues using Trizol according to the manufacturer's instructions (Invitrogen). cDNA was synthesized using random primer and a High Capacity cDNA Reverse Transcription Kit (Applied Biosystems). The primer pairs used for analysis of cDNA were showed in Table 2 [52], [53], [54]. Quantitative real-time PCR was performed on the 7500 Real Time PCR System (Applied Biosystems) with a program of 50°C for 2 min, 95°C for 10 min and 40 cycles of 95°C for 15 s; 60°C for 1 min. Template copy numbers for PCR cycle thresholds were extracted using standard graphs. For each sample, template copy numbers were internally normalized with their respective input control. Relative expression was calculated as the ratio of template copy numbers of a sample relative to the negative control after normalizing with their respective isotype control Actin and is shown as the mean ± SD. Statistical significance was determined by a one-tail paired Student's t test.

**Table 2:** Primer sequences used in real-time quantitative PCR.

Sequence no.	Gene name	Primer sequence	Accession no.	Tm (°C)	Product size (bp)	Reference
1	Bax	F- ATGGAGCTGCAGAGGATGATTR- TGAAGTTGCCATCAGCAAACA	NM017059	60	97	Perez et al [54]
2	Bcl-2	F- TGGGATGCCTTTGTGGAACTR-TCTTCAGAGACTGCCAGGAGAAA	U34964	60	73	Perez et al [54]
3	caspase-3	F- AATTCAAGGGACGGGTCATGR- GCTTGTGCGCGTACAGTTTC	U49930	60	67	Kijima et al [53]
4	GRP78	F- AACCCAGATGAGGCTGTAGCAR- ACATCAAGCAGAACCAGGTCAC	NM022310	60	91	Liu et al [52]
5	CHOP	F- CCAGCAGAGGTCACAAGCACR- CGCACTGACCACTCTGTTTC	NM007837	60	126	Liu et al [52]
6	JNK	F- TGATGACGCCTTACGTGGTAR- GGCAAACCATTTCTCCCATA	XM341399	60	114	Liu et al [52]
7	caspase-12	F- CACTGCTGATACAGATGAGGR- CCACTCTTGCCTACCTTCC	NM130442	60	138	Liu et al [52]
8	Actin	F- TTGCTGATCCACATCTGCTGR- GACAGGATGCAGAAGGAGAT	AB028846	60	146	Kijima et al [53]

### Western blot analysis

A small amount of kidney tissue were ground under liquid nitrogen, the power was quickly mixed with a lysis buffer containing 7 M urea, 2 M Thiourea, 4% Chaps, 1% DTT, 40 mM Tris-base, 1 mM PMSF. Equal amounts of protein from each sample were boiled in SDS sample buffer for 10 min. SDS gel electrophoresis and electroblotting on polyvinylidene fluoride (PVDF) membrane were based on the work of Towbin et al [55]. Briefly, following separation on a 12% separation gel and 5% stacking gel by electrophoresis, the separated proteins were transferred electrophoretically onto a PVDF membrane, which was then blocked with 5% skim milk. The PVDF membranes were incubated for 1 h with one of the following antibodies: GRP78, caspase-12, CHOP, JNK (WUHAN BOSTER BIO-ENGINEERING LIMITED COMPANY; Beijing Biosynthesis Biotechnology Co.,LTD, China) diluted 1∶400 in 5% skim milk. After being washed, the membranes were incubated with the corresponding secondary antibody conjugated to horseradish peroxidase (Proteintech; 1∶4000 in 5% skim milk). The conjugated substrate was detected using a chemiluminescent dectection kit (CWBIO, China). Equal protein loading was confirmed by staining with beta-actin antibody (WUHAN BOSTER BIO-ENGINEERING LIMITED COMPANY, China).

### Quantitative analysis and statistical analysis

Experimental data were analyzed by one-way ANOVA using SAS statistical program (SPSS Institute Inc., Cary, NC, USA). The results were expressed as means and standard errors. Differences were considered to be statistically significant with p<0.05 indicated as one asterisk and P<0.01 as double asterisks.
